# Association between glomerular filtration rate (measured by high-performance liquid chromatography with iohexol) and plasma oxalate

**DOI:** 10.1590/1678-4685-JBN-3743

**Published:** 2018-04-09

**Authors:** Luciano da Silva Selistre, Pierre Cochat, Dener lizot Rech, François Parant, Vandréa Carla de Souza, Laurence Dubourg

**Affiliations:** 1 Universidade de Caxias do Sul, Caxias do Sul, Brasil.; 2 Université Claude-Bernard Lyon, Centre de Référence des Maladies Rénales Rares Nephrogones, Service de Néphrologie et Rhumatologie Pédiatriques, Lyon, France.; 3 Hospices Civils de Lyon, GHS - Centre de Biologie Sud, UM Pharmacologie - Toxicologie, F-69495, Pierre Bénite, France.; 4 Université Claude-Bernard, Groupement Hospitalier Edouard Herriot, Hospices Civils de Lyon, UMR 5305, Rhone-Alpes, Lyon, France.

**Keywords:** Glomerular Filtration Rate, Hyperoxaluria, Chronic kidney disease, Taxa de filtração glomerular, Hiperoxalúria, Doença renal crônica

## Abstract

**Introduction:**

Secondary hyperoxalemia is a multifactorial disease that affects several organs and tissues in patients with native or transplanted kidneys. Plasma oxalate may increase during renal failure because it is cleared from the body by the kidneys. However, there is scarce evidence about the association between glomerular filtration rate and plasma oxalate, especially in the early stages of chronic kidney disease (CKD).

**Methods:**

A case series focuses on the description of variations in clinical presentation. A pilot study was conducted using a cross-sectional analysis with 72 subjects. The glomerular filtration rate (GFR) and plasma oxalate levels were measured for all patients. Results: Median (IQR) GFR was 70.50 [39.0; 91.0] mL/min/1.73 m^2^. Plasma oxalate was < 5.0 µmol/L in all patients with a GFR > 30 mL/min/1.73m^2^. Among the 14 patients with severe CKD (GFR < 30 mL/min/1.73 m^2^) only 4 patients showed a slightly increased plasma oxalate level (between 6 and 12 µmol/L).

**Conclusion:**

In non-primary hyperoxaluria, plasma oxalate concentration increases when GFR < 30mL/min/1.73 m^2^ and, in our opinion, values greater than 5 µmol/L with a GFR > 30 mL/min/1.73 m^2^ are suggestive of primary hyperoxaluria. Further studies are necessary to confirm plasma oxalate increase in patients with low GFR levels (< 30mL/min/1.73 m^2^).

## RESUMO

**Introdução:**

A hiperoxalemia secundária é uma doença multifatorial que afeta vários órgãos e tecidos em pacientes com rins nativos ou transplantados. O oxalato plasmático pode aumentar durante a insuficiência renal porque é eliminado do corpo pelos rins. No entanto, há evidências escassas sobre a associação entre taxa de filtração glomerular e oxalato plasmático, especialmente nos estágios iniciais da doença renal crônica (DRC).

**Métodos:**

uma casuística centrada na descrição das variações na apresentação clínica. Foi realizado um estudo piloto a partir da análise transversal com 72 indivíduos. As taxas de filtração glomerular (TFG) e os níveis plasmáticos de oxalato foram medidos para todos os pacientes. Resultados: A TFG mediana (IIQ) foi de 70,50 [39,0; 91,0] mL/min/1,73 m^2^. O nível plasmático de oxalato foi < 5,0 µmol/L em todos os pacientes com TFG > 30 mL/min/1,73 m2. Entre os 14 pacientes com DRC grave (TFG < 30 mL/min/1,73 m^2^), apenas quatro apresentaram ligeiro aumento do nível plasmático de oxalato (entre 6 e 12 µmol/L).

**Conclusão:**

Na hiperoxalúria não primária, a concentração plasmática de oxalato aumenta quando TFG < 30 mL/min/1,73 m^2^ e, em nossa opinião, valores superiores a 5 µmol/L com TFG > 30 mL/min/1,73 m^2^ sugerem presença de hiperoxalúria primária. Estudos adicionais são necessários para confirmar o aumento do oxalato plasmático em pacientes com níveis baixos de TFG (< 30 mL/min/1,73 m^2^).

## INTRODUCTION

Oxalate, the ionic form of plasma oxalate (POx), is an insoluble end-product of the metabolism of foods derived from various animal and plant sources. Increase of POx and oxalosis, i.e., calcium oxalate deposition in tissues, can result in primary (PH) or secondary hyperoxaluria (SH).^1^^,^ 2 PHs are a group of rare autosomal recessive metabolic disorders resulting in overproduction of oxalate,^3^ caused by the deficiency of three different enzymes and affecting a different intracellular organelle;^1^^,^ 2 the disorders are designated as PH types 1, 2, and 3.

SH may occur either as a result of excessive ingestion of oxalate or oxalate precursors such as ethylene glycol or through decreased excretion of oxalate by the kidney.^1^ Excessive intake or increased absorption (intestinal disorders) of oxalate are widespread causes of urinary oxalate excretion increase and urolithiasis, but is rarely associated to an increase of POx and occurs under normal glomerular filtration rate (GFR).^3^^-^ 5 Thus, it cannot be further processed and is eliminated through normal filtration by the kidneys. In patients with chronic kidney disease (CKD), POx accumulates 10-30 times above normal levels as a result of its reduced excretion. Neither hemodialysis nor peritoneal dialysis can normalize POx levels in CKD patients; a 60% reduction is expected after a usual hemodialysis procedure, but POx was found to return to pre-dialysis levels within 48 h.^7^ In contrast to PH, clinical manifestations of uremic oxalosis, such as nephrolithiasis, fractures, and bone pain, are uncommon.^1^^,^ 7

Nevertheless, a decline in GFR can increase POx and lead to cardiovascular complications.^1^^,^ 2 ^,^ 6 ^,^ 7 Salye *et al.* reported that SH, i.e. renal and myocardial calcium oxalate deposition, in association with renal insufficiency is frequent and often extensive.^8^ In addition, they demonstrated that the incidence and severity of the oxalate deposition are related to the duration of renal insufficiency.^8^^,^ 9 Rechet *et al.* described an association between high levels of POx and endothelial injury leading to atherogenic effects by elevating intracellular calcium in endothelial cells.^7^ In transplanted kidneys, POx may overload the graft with potential tubular damage, affecting function.^3^^,^ 6 ^,^ 11

Therefore, we designed a pilot study with measured POx, measured GFR (mGFR, iohexol clearance), and estimated GFR (eGFR, plasma creatinine) in a series of patients with various stages of CKD.

## MATERIALS AND METHODS

### PATIENTS

For this pilot study, 72 CKD patients without PH were recruited between October 2014 and November 2014 to undergo GFR measurement (iohexol clearance). Ten patients presented with a severe CKD (stage IV-V) and POx without an mGFR. The sample was divided into groups according to GFR following the KDIGO classification.^12^

### OXALATE

POx was measured on a Pentra 400 analyzer (HORIBA) by a modified sensitive oxalate oxidase colorimetric assay as reported by Petrarulo *et al*. Briefly, oxalate is converted to hydrogen peroxide, which, in the presence of peroxidase, reacts (POD) with MBTH (3-methyl-2-benzothiazolinone hydrazone) and DMAB (3-dimethylamino benzoic acid) forming a blue quinone compound. The color intensity is proportional to the concentration of POx in the sample and is read at 600 nm with 700 nm as reference wavelength. The optimization of the assay included plasma deproteinization by the sulfosalicylic acid (SSA) method and treatment with charcoal. The reference values are < 5 µmol/L.^13^

### GFR MEASUREMENT BY IOHEXOL CLEARANCE

Iohexol clearance was performed using a standard technique with single-bolus injection. Briefly, an i.v. injection of 6 mL (Omnipaque, 300 mg/mL) was administered and 3 blood samples were drawn from the contra lateral arm after 120, 180, and 240 minutes. The mGFR was calculated from the slope of plasma concentrations using a one-compartment model corrected with the Bröchner-Mortensen formula.^13^ Plasma iohexol concentration was determined with an HPLC method adapted from Cavalier *et al*.^15^ The results were reported in mL/min/1.73 m^2^.

### ESTIMATION OF GFR

Plasma creatinine (PCr) was measured by an IDMS-standardized enzymatic method, and eGFR was calculated with the CKD-EPI equation.^12^

### STATISTICAL ANALYSES

We evaluated the distribution of continuous variables by calculating mean ± standard deviation and categorical variables by number (percentage) in the whole data set, as well as in subgroups according to study population characteristics and candidates for living kidney donation. The error was calculated by subtracting eGFR from mGFR minus (mGFR - eGFR) for each individual; percent error was this difference relative to mGFR, i.e., (mGFR - eGFR)/mGFR. We computed the bias as the average error, which was appropriate for the distribution.

Bias, an expression of systemic error in estimated GFR, is defined as the median or mean of the differences between estimated and measured GFR. The analysis was performed using R for windows, version 3.1.1 (*R-Cran project*, http://cran.r-project.org/).

### ETHICAL APPROVAL

All the procedures were carried out in accordance with the ethical standards of the institutional and/or national research committees and of the 1964 Helsinki Declaration and its later amendments or comparable ethical standards. An appropriate informed consent was signed by all the participants or their legal representatives. The consent form contained information on the procedure itself as well as on the possible use of the data for research purposes. According to the current French laws, an observational study that does not change routine management of patients does not need to be declared or submitted to a research ethics board (Loi Huriet-Sérusclat 88-1138, 20 December 1988 and its subsequent amendments, text available at http://www.chu-toulouse.fr/IMG/pdf/loihuriet.pdf).

## RESULTS

The patient clinical characteristics are listed in the Table 1. The mean age and BMI were 50.0 [IQR, 40.0-63.0] years and 25.3 [IQR, 22.3-32.0] kg/m^2^, respectively.

**Table 1:** Characteristics of the patients

Number of participants	72
Age (years)	50 [40; 63]
Male	40 (56.3)
Diagnosis	
CKD	60 (83)
Candidate for living kidney *donation*	12 (17)
Weight (kg)	72.0 [60.5; 90.0]
Height (cm)	168.0 [160.0; 174.5]
Body surface area (m^2^)	1.83 [1.63; 2.01]
Body mass index (kg/m^2^)	25.3 [22.3; 32.0]
mGFR (mL/min/1.73 m^2^) (n = 62)	74.5 [53.0; 91.0]
eGFR (mL/min/1.73 m^2^)	70.0 [39.0; 96.0]
mean mGFR-eGFR (95%CI), mL/min/1.73 m^2^ (N = 62)	-3.8 (-7.0; -0.5)
Plasma oxalate	
Plasma oxalate < 5 µmol/L	67 (93.0)
Plasma oxalate ≥ 5 µmol/L	5 (7.0)
CKD stages	
Stage I	16 (22.2)
Stage II	28 (38.9)
Stage IIIa	4 (5.5)
Stage IIIb	10 (13.9)
Stage IV	11 (15.3)
Stage V	3 (4.0)

Values are median (IQR) or n (%) unless otherwise specified CKD: chronic kidney disease; GFR: glomerular filtration rate; IQR: interquartile range. CKD stages were determined according to mGFR or eGFR if not available.

**Figure 1:**
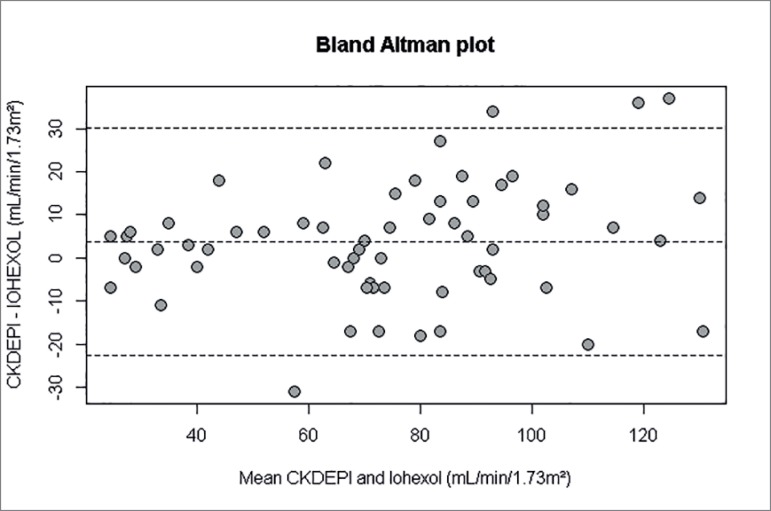
Glomerular filtration by iohexol and age (years) of the study participants.

The Table 1 and Figure 1 show the performance of the CKD-EPI equation versus iohexol. CKD-EPI equation had a mean error of -3.0 (95%CI, -7.0 to -0.5) mL/min/1.73 m^2^ without statistical difference to GFR. Therefore, the use of CKD-EPI in patients without mGFR could be adequate for evaluation of POx levels.

For all but one patient with mGFR, the POx concentration was < 5µmol/L. One patient had abnormal values of POx (7 µmol/L) with a mGFR of 30 mL/min/1.73 m^2^. Among the 14 patients with severe CKD (GFR < 30 mL/min/1.73 m^2^), four patients showed a slightly increased POx (between 6 and 12 µmol/L) (Table 1).

## DISCUSSION

POx can increase in CKD due to the reduced GFR and secretion of the proximal renal tubules.^1^^,^ 2 ^,^ 15 ^-^ 18 However, few authors have specifically studied the correlation between POx and GFR stage.^7^^,^ 15 ^-^ 20 In addition, some authors established a POx threshold level that differentiate PH from other causes of POx increase.^1^^,^ 2

Constable *et al*. reported that POx was raised by a factor of 10 in PH subjects who still had good renal function.^18^ By contrast, Morgan *et al*. demonstrated in patients with non-PH-CKD that oxalate retention is increased when GFR is below 20 mL/min/1.73m^2^.^20^ In the same way, Constable *et al*. reported that the oxalate metabolic pool expands rapidly when the GFR is under 25 mL/min/1.73 m^2^, which is in accord with our results. Barratt *et al*. found that POx is also increased in end stage renal disease (ESRD).^19^ Bhasin *et al*. reported that POx was higher than 80 µmol/L in PH patients with ESRD.^1^^,^ 2 Elgstoen *et al*. found that median POx before transplantation was 35.0 µmol/L (95%CI: 10.4 to 93.9) and 98% of the values were above normal limits.^11^

In the present study, we found a slightly increased POx with GFR < 30 mL/min per 1.73 m^2^, well above the level at which renal replacement is needed. However, we were not able to demonstrate a correlation between GFR and POx.

The strengths of the present study are i) the reference method (iohexol) for direct measurement of GFR for most of the patients; and ii) the wide ranges for GFR levels (7 to 139 mL/min/1.73 m^2^).

Study limitations are: i) the population sample included few patients with GFR < 30 mL/min/1.73 m², which did not allow establishing a correlation between POx and GFR.

## CONCLUSION

This study suggests that POx increases significantly only in advanced stages of CKD. In our opinion, values greater than 5 µmol/L with an eGFR > 30 mL/min/1.73 m^2^ are suggestive of PH. However, new studies should determine the kinetics of POx in advanced CKD and dialysis patients.
